# Spatial Variability in the Effect of High Ambient Temperature on Mortality: An Analysis at Municipality Level within the Greater Athens Area

**DOI:** 10.3390/ijerph16193689

**Published:** 2019-09-30

**Authors:** Sofia Zafeiratou, Antonis Analitis, Dimitra Founda, Christos Giannakopoulos, Konstantinos V. Varotsos, Panagiotis Sismanidis, Iphigenia Keramitsoglou, Klea Katsouyanni

**Affiliations:** 1 Department of Hygiene and Epidemiology, University of Athens Medical School, GR11527 Athens, Greece; 2 Institute for Environmental Research and Sustainable Development, National Observatory of Athens, GR15236 Athens, Greece; 3 Institute for Astronomy, Astrophysics, Space Applications and Remote Sensing, National Observatory of Athens, GR15236 Athens, Greece; 4 School of Population Health & Environmental Sciences, King’s College London, London SE1 9NH, UK

**Keywords:** heat-mortality association, intra-city variability, effect modifiers, socioeconomic modifiers, environmental modifiers

## Abstract

Spatial variability in temperature exists within metropolitan areas but very few studies have investigated intra-urban differentiation in the temperature-mortality effects. We investigated whether local characteristics of 42 Municipalities within the Greater Athens Area lead to modified temperature effects on mortality and if effect modifiers can be identified. Generalized Estimating Equations models were used to assess the effect of high ambient temperature on the total and cause-specific daily number of deaths and meta-regression to investigate effect modification. We found significant effects of daily temperature increases on all-cause, cardiovascular, and respiratory mortality (e.g., for all ages 4.16% (95% CI: 3.73,4.60%) per 1 °C increase in daily temperature (lags 0–3). Heterogeneity in the effect estimates between Municipalities was observed in several outcomes and environmental and socio-economic effect modifying variables were identified, such as % area coverage of buildings, length of roads/km^2^, population density, % unemployed, % born outside the EU countries and mean daily temperature. To further examine the role of temperature, we alternatively used modelled temperature per Municipality and calculated the effects. We found that heterogeneity was reduced but not eliminated. It appears that there are socioeconomic status and environmental determinants of the magnitude of heat-related effects on mortality, which are detected with some consistency and should be further investigated.

## 1. Introduction

Several studies have provided evidence that high temperatures and heat waves are strongly associated with adverse effects on all-cause and cause-specific mortality [1,2,3,4,5,6,7,8]. There is also evidence that there are vulnerable groups such as the elderly [9,10,11,12,13].

The association between daily mortality and temperature on the same or a few preceding days is a U-shape association and that has been observed in many parts of the World [2,6,14]. There is an optimum temperature value (“threshold” temperature) at which minimum mortality is observed whilst mortality increases with temperatures lower or higher than this point. There are geographical differences in the specification of this U-shaped curve. Locations with warmer climates have higher temperature threshold values while the threshold is lower in colder climates [2,14]. This phenomenon may be a result of adaptation of the population to the local climate or may be driven by different exposure patterns determined by cultural or social characteristics [2,14]. 

Today, more than half of worlds’ population are urban residents and the percentage is projected to increase in the near future. Although increasing urbanization and modification of urban climates is one of the most important topics in the current century, the recognition of the effect of urban changes on the natural environment and local climates dates back to the 19th century, with the observations of Luke Howard in the city of London [15]. In the following decades, the study of urban climates received special attention worldwide, and published research shed light on the factors that modulate urban climate variability and form ‘Urban Heat Islands’ (UHI) that make cities hotter than surrounding non-urban environments [16,17,18,19]. Modification of surface characteristics and surface energy budget represent the main contributing components that modulate urban local climates. Such modifications are mainly associated with increased heat storage from built surfaces, decreased latent and sensible heat exchange (and thus reduction of evaporative cooling) and heat added by human activities in the city [17]. Under climate change, an increase in temperature and in the frequency and intensity of heat waves is expected and thus heat-related health effects have attracted more attention recently [20]. Heterogeneity between different cities or areas with different climatic conditions in temperature effects on mortality has also been studied [6,21]. Furthermore, there is some evidence that heat-wave related mortality is more pronounced in urban compared to rural areas, due to the UHI effect [22,23]. 

Since the UHI is determined by urban planning and land use variables, it may be expected that its intensity is not homogeneously distributed over a large metropolitan area. However, there are limited studies investigating the spatial variability of the effects of temperature on mortality within a metropolitan area. Ying Yang Chan et al. [24] and Goggins et al. [25,26] investigated the effect of UHI and Socio-Economic Status (SES) within Hong-Kong and Kaohsiung, Taiwan, Smargiassi et al. [27] studied the effects of UHI in Montreal Canada, Vaneckova et al. [28] studied the spatial variability of mortality among the elderly in Sydney Australia, Milojevic et al. [29] studied the UHI effects in London, Hattis et al. [30] in Massachussets, Klein Rosenthal et al. [31] and Madrigano et al. [32] in New York City, whilst Vargo et al. [33] assessed the impact from UHI reduction policies in three U.S. metropolitan areas and Carmona et al. [34] in Madrid. Some of the studies also investigated how temperature effects differ between city areas with different urban landscape and/or socio-demographic characteristics [35]. To determine the spatial variation of temperature within a city and assess the presence of the UHI, some studies relied on neighborhood, urban design or other area characteristics [24,25,26] whilst some used satellite data providing land surface temperature across a city [27,31]. One study was based on modelling temperature across London [29] and one other study used kriging based on a large number of monitors [30]. There is substantial variability in the approaches and a lack of studies within metropolitan areas in Europe. 

In the present study, within the EU funded TREASURE (Thermal Risk rEduction Actions and tools for SecURE cities; ECHO/SUB/2014/695561) and EXTREMA (EXTRemetEMperature Alerts for Europe;DG ECHO, 2018-2019, GA 783180) projects [36], we investigated whether characteristics of Municipalities within the Greater Athens Area in Greece, a metropolitan area with more than 3 million inhabitants, leads to modified temperature effects on mortality of the area residents and if effect modifiers can be identified.

## 2. Materials and Methods 

### 2.1. Study Area

Data for the Area (438.70 km^2^) were used. The Greater Athens Area is surrounded by four mountains and is open to the sea on the Southwest (Saronic Gulf). The major axis of the valley runs from north-east to south-west for about 30 km. Most industry lies close to the sea and the harbour of Piraeus in the south-western part of Athens area. The climate of Athens is typically Mediterranean (Köppen climate classification: Csa), characterized by hot and dry summers and rainy winters. Based on the centennial climatic records of the National Observatory of Athens (NOA), the long-term climatic value of the average annual temperature is 17.7 °C and about 400 mm of precipitation falls annually. The mean daily temperature during the winter months is 10.1°C and the lowest average temperature in the year occurs in January, when it is around 9.5 °C. During the summer months, the mean daily temperature is 26.1 °C and the mean value of the maximum daily temperature is about 31.5 °C. At an average temperature of 27 °C (mean maximum temperature of 33 °C), July is the hottest month of the year [32]. The prevailing wind direction is north-north-east at the end of summer, in autumn and in winter, and south-south-west in spring and the beginning of the summer [37,38]. Athens has been experiencing a significant and ongoing warming during the last few decades and particularly since the mid-1970s, which is more pronounced in summer, amounting to nearly 0.7 °C/decade. This warming is accompanied by simultaneous increase in the frequency of hot days and heat waves, as well as in the frequency of heat-related thermal discomfort in the city [39]. Moreover, capital cities of the eastern Mediterranean like Athens have been characterized as hot spots with respect to future heat stress among 571 European cities [40].

**Figure 1:**
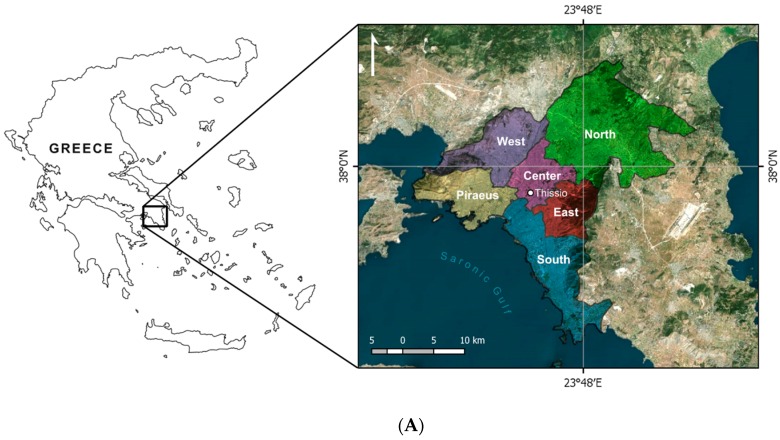

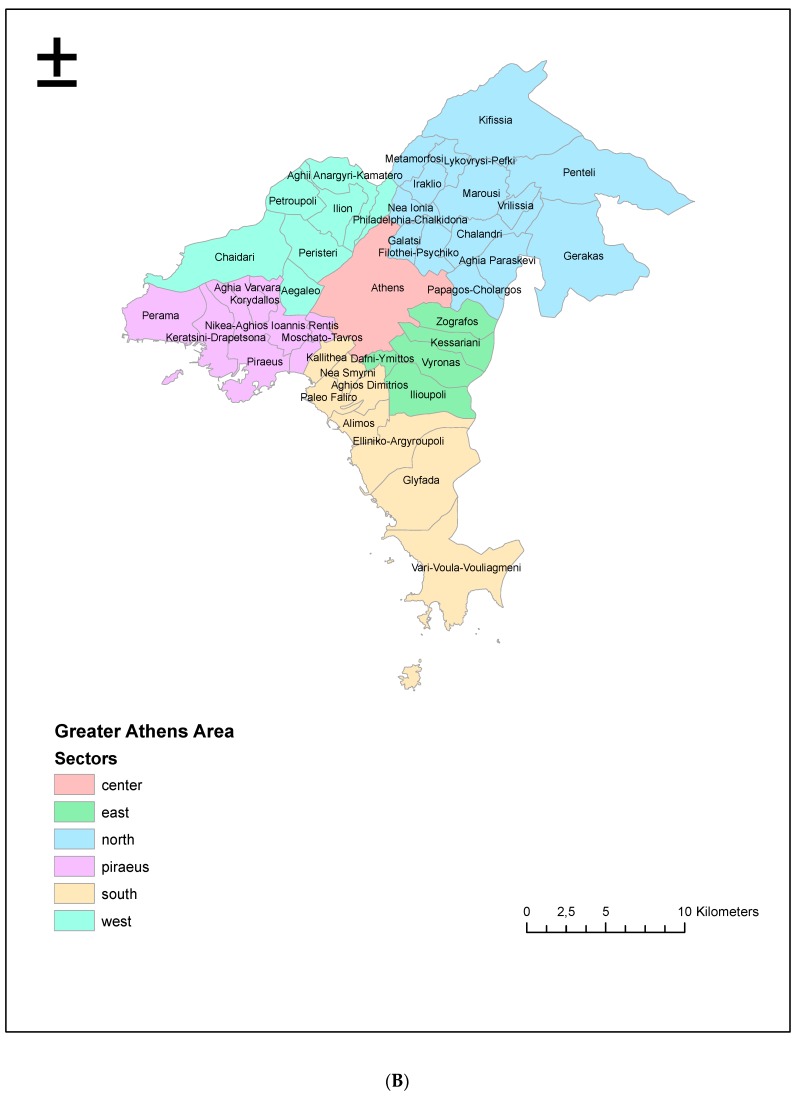
(**A**) Map of Greece and the Athens area showing the subdivision into six sectors. (**B**) The Greater Athens area with the subdivision into six Sectors and 42 Municipalities.

The analysis was restricted to the warm period (from April to September) of the years 2000–2012. The Greater Athens Area is divided into 42 Municipalities which may be classified into six sectors (Athens center, Piraeus, North, South, West, and East; Figure 1A,B), based on geographical criteria.

### 2.2. Mortality Data

Data on the daily number of deaths among residents of each Municipality were provided by the Hellenic Statistical Authority. We used the daily number of deaths caused by all natural (ICD-9: 1–799; ICD-10: group A–R), cardiovascular (ICD-9: 390–459; ICD-10: group I) and respiratory (ICD-9: 460–519; ICD-10: group J) causes, for all ages and for age categories 65–74 and 75+ years. 

### 2.3. Meteorological Data

Meteorological data of air temperature (°C) (daily mean and maximum) and relative humidity (%) were provided by the National Observatory of Athens (NOA). These data were measured at a fixed-site station located in Thissio (Lat.: 38°0.00′ N, Lon.: 23°43.48′ E), in the center of Athens. To characterize the geographical variability of temperature we also used temperature data obtained from the E-OBS dataset, developed within the ENSEMBLES project for the six sectors separately [41,42,43]. Specifically, E-OBS covers the entire European land surface and is based on the “European Climate Assessment and Dataset” station data set, plus more than 2000 further stations from different archives. A three-step process of interpolation to the station data is employed, by first interpolating the monthly precipitation totals and monthly mean temperature using three-dimensional thin-plate splines, then interpolating the daily anomalies using indicator and universal kriging for precipitation and kriging with an external drift for temperature, then combining the monthly and daily estimates. Gridded datasets, derived through interpolation of station data, have a number of potential uncertainties and errors. These can be introduced either by the propagation of errors in the station data or by limitations in the ability of the interpolation method to estimate grid values from the underlying station network. As part of the ENSEMBLES project, MeteoSwiss has evaluated the homogeneity of the station data [42] and Oxford University has evaluated the gridded E-OBS dataset [43]. An important finding of the latter evaluation is that, in areas where relatively few stations have been used for the interpolation, both precipitation and temperature are over-smoothed. This leads to reduced interpolated values relative to the “true” area-averages, in particular for extremes.

In our analysis, we used the maximum hourly temperature per day, averaged over 4 days: the same day and the 3 previous ones (lags 0–3).

### 2.4. Effect Modifiers and Confounders

To investigate the spatial variability of temperature effects on mortality within the metropolitan area of Athens, Municipality-level environmental and Socio-Economic Status characteristics were used as potential effect modifiers. Specifically, population density (number of inhabitants per km^2^), area covered by green space (%), density of the road network within municipality (total road length in km perkm^2^), area covered by buildings (%), total area (km^2^), population born in non-EU28 countries (%), unemployment rate (%), youth unemployment rate (%), long-term unemployment rate (12 months and more, %), population aged 25–64 with lower secondary education attainment (%), population aged 25–64 with upper secondary or tertiary education attainment (%), early leavers from education and training (%) were considered as effect modifiers. The SES variables concerning the origin of the population, variables related to aspects of unemployment and education are not so relevant for the age groups having by the highest mortality. However, they characterize the general SES of an area which affects the whole of the area population. Data were provided by the Hellenic Statistical Authority, except area covered by green space which was obtained from the European Urban Atlas 2012 dataset. Additionally, the average of the mean and the maximum temperature from the E-OBS model was used as potential effect modifier. Day of the week, seasonality and long-term trends (over the whole period 2000-12) were used as confounders.

### 2.5. Statistical Analysis

#### Temperature-Mortality Association 

As mentioned before, the daily temperature-mortality association curve is known to be U-or J-shaped. Since our analysis included only the warm period, we expect to obtain a rather J-shaped curve, where the turning point is the temperature associated with the lowest mortality, i.e., the “threshold” temperature. We described this temperature-mortality curve using two linear terms, constrained to join at the threshold temperature. We used the series of measurements from the central meteorological site “Thissio” which reflects the temporal variation of the daily temperature well, but not the spatial. There is heterogeneity between areas in the observed temperatures but there are not enough meteorological stations to characterize spatial variability. Thus, the values measured will potentially underestimate the real temperature in some Municipalities or sectors (the “hotter” ones) and overestimate it in others (the “cooler” ones). In this situation, we would expect differentiation in the thresholds as well as the effect estimates above the threshold in the various areas, depending on their local characteristics.

We first estimated the temperature threshold, above which mortality increases, for the whole of the Greater Athens Area, using the temperature series from Thissio station. Muggeo’s method was used for the threshold estimation [44]. After the estimation of the threshold, we used Generalized Estimating Equations (GEE) models to assess the effect of high temperature on the total and cause-specific daily number of deaths. The model was of the form:

log E[Yij] ~ β_1_×T_+_ + β_2_×T_−_ + Σβ_k_×dow + Σβ_m_×month + f(time) (1)

where Yij is the daily number of deaths on day i of year j, assumed to follow a Poisson distribution with expectation μ, and T_+_ and T_−_ are the average 4-day temperature (lags 0–3) terms above and below the threshold value. In the model we also included, six indicator variables for day of week (dow), five indicator variables for month and a quadratic term for time (from 1^2^ to 2379^2^), to adjust for seasonality and long term trends. A first order correlation structure (AR1) was specified, based on previous results [2]. The analysis was carried out for each cause of death and for each age group, for the Greater Athens area, for the 42 municipalities and for the six sectors in which they were classified, using the Thissio meteorological data. 

The heterogeneity in the effect estimates of heat on mortality in the 42 municipalities was assessed using I^2^ index. When statistically significant heterogeneity was evident, we applied univariate meta-regression models, using the previously presented variables that characterized each municipality. We also used the mean value of 24-h average and maximum temperature from the E-OBS database for 2000–2012 for the corresponding sector (°C) as potential effect modifier. To illustrate effect modification, we calculated the temperature effect at the 25th and the 75th percentile of the distribution of the corresponding effect modifier. 

To further investigate the impact of the geographical differentiation in temperature effect and the extent to which the potentially identified effect modifiers operate through an association with temperature itself, we applied a second approach in which we used the temperature as estimated in each Sector by the E-OBS data series. This is assumed to approximate the real conditions faced by inhabitants better. With this approach, we would expect less differentiation between effect estimates. In this approach, the temperature threshold was estimated for each sector separately (sector-specific threshold). 

In all approaches, the effect of temperature on mortality was calculated as percentage increase in mortality per °C increase above the threshold. 

Stata version 13 ( StataCorp LP, College Station, TX, USA) [45] and R software (R Foundation for Statistical Computing, Vienna, Austria) [46] were used for the statistical analysis.

## 3. Results

The average value of the daily mean and daily maximum temperature using the Thissio station measurements for the study period was 24.5 °C and 29.1 °C respectively (Table 1). Based on E-OBS data the average mean temperature varied from 21.8 °C at West and North sector to 23.4 °C at Athens center, whilst the maximum temperature varied from 26.1 °C at West sector to 27.7 °C at Athens center (Table 1). The highest temperature values were observed in July and lowest in April (data not shown). It can be seen that the temperature measurements from Thissio station were higher by 1–2 °C on the average than the modelled E-OBS temperature. The average daily relative humidity was 54%, based on measurements at Thissio station.

In the Greater Athens Area, the average daily number of deaths was 77, 35 and 7 from all natural, cardiovascular and respiratory causes, respectively (Table 2). Sixty six percent of the deaths were observed in people over 75 years. The population by sector ranged from 270,573 inhabitants (east sector) to 706,250 inhabitants (north sector) (Table 2) and by municipality from 25,389 (Perama municipality, Piraeus sector) to 664,046 inhabitants (Athens municipality, Athens center). 

**Figure 2:**
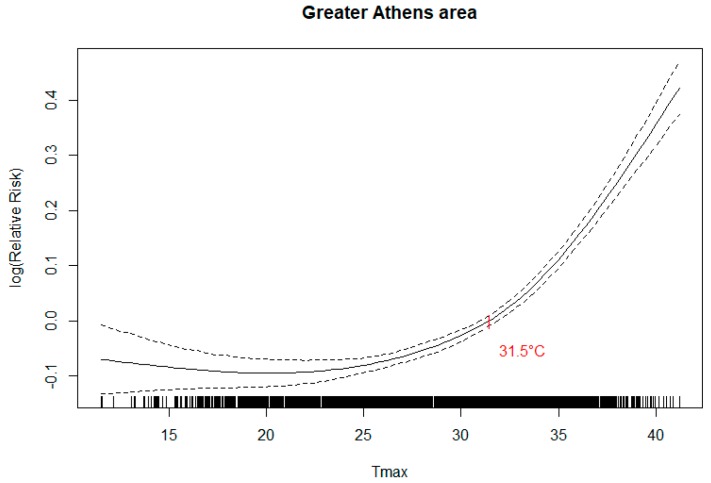
Temperature-mortality association (dotted line: 95 % Confidence Interval) for the Greater Athens Area, using the Thissio measurements.

The association curve between temperature and all natural cause mortality for the whole of the Greater Athens Area is shown in Figure 2. The same association per sector using the E-OBS data is shown in the supplementary material, Figure S1. The estimated temperature threshold was 31.5 °C for Greater Athens Area when the Thissio data were used and varied from 24.8 °C in the west sector to 27.5 °C in the Athens center when the E-OBS sector-specific thresholds were estimated.

Table 3 presents the percent increase in mortality per 1 °C increase in maximum temperature (average of lags 0–3) above the estimated threshold (31.5 °C), by cause of death and age in the Greater Athens Area. For all ages there was a 4.16% (95 % CI: 3.73, 4.60 %) increase in all natural causes mortality, 5.34 % (95 % CI: 4.74, 5.93 %) in cardiovascular mortality and 5.90% (95 % CI: 5.57, 7.24) in respiratory mortality. The largest increase in mortality was estimated in the 75+ age group for all natural and cardiovascular mortality (5.17 %, 95 % CI: 4.65, 5.69 and 6.17 %, 95 % CI: 5.49, 6.86 respectively) and in the 65–74 age group for respiratory mortality (7.68 %, 95 % CI: 4.39, 11.08). The results remained very close (change in % increase < 10 %) when relative humidity was included in the models in addition to temperature.

**Figure 3:**
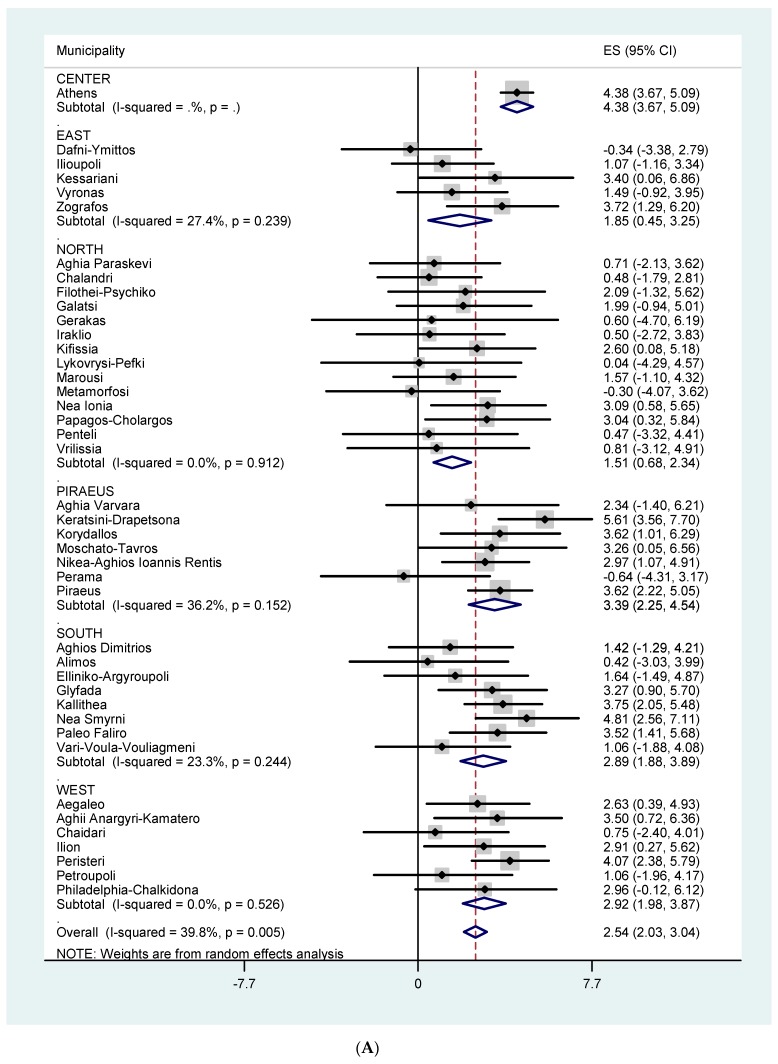

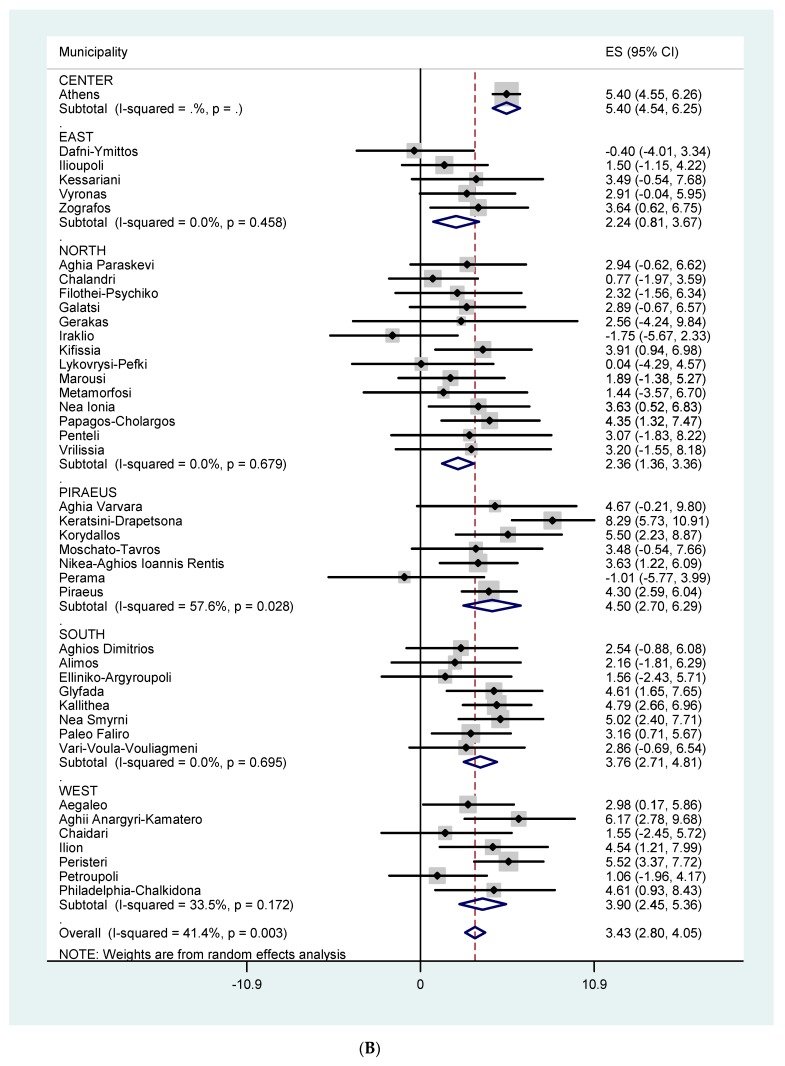
(**A**) % increase in total mortality, all ages, per 1 °C increase in maximum temperature in each municipality, using Thissio measurements and threshold of 31.5 °C. (**B**) % increase in total mortality among the elderly (75+) per 1 °C increase in maximum temperature in each municipality, using Thissio measurements and threshold of 31.5 °C.

**Figure 4:**
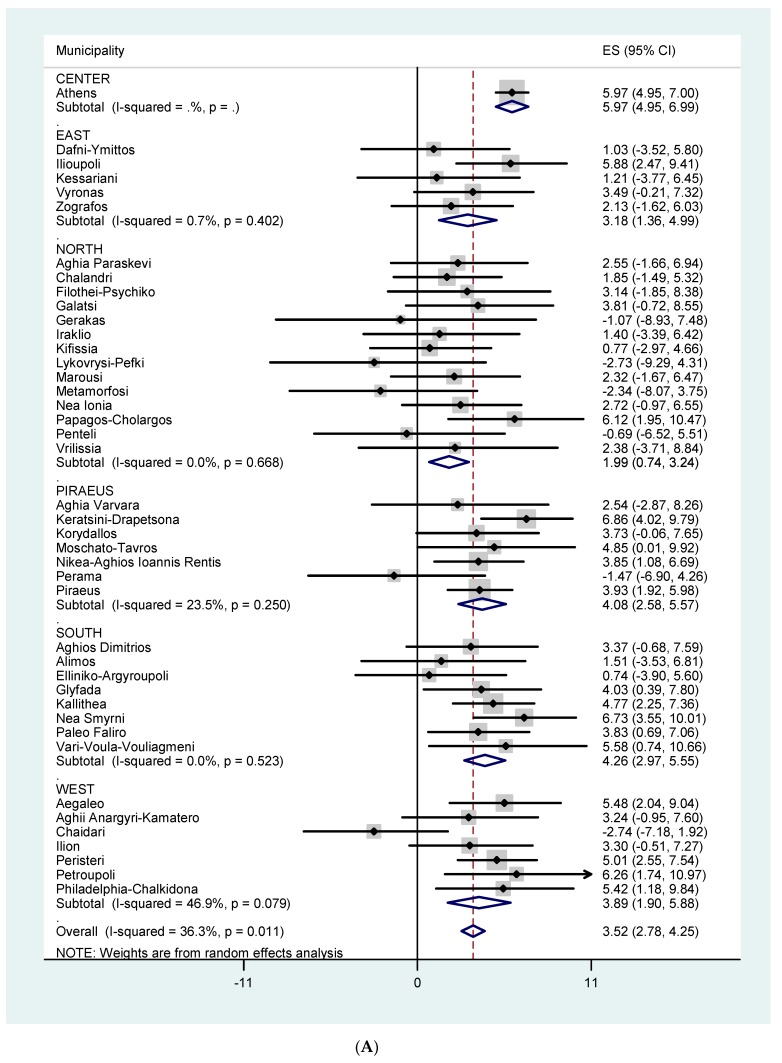

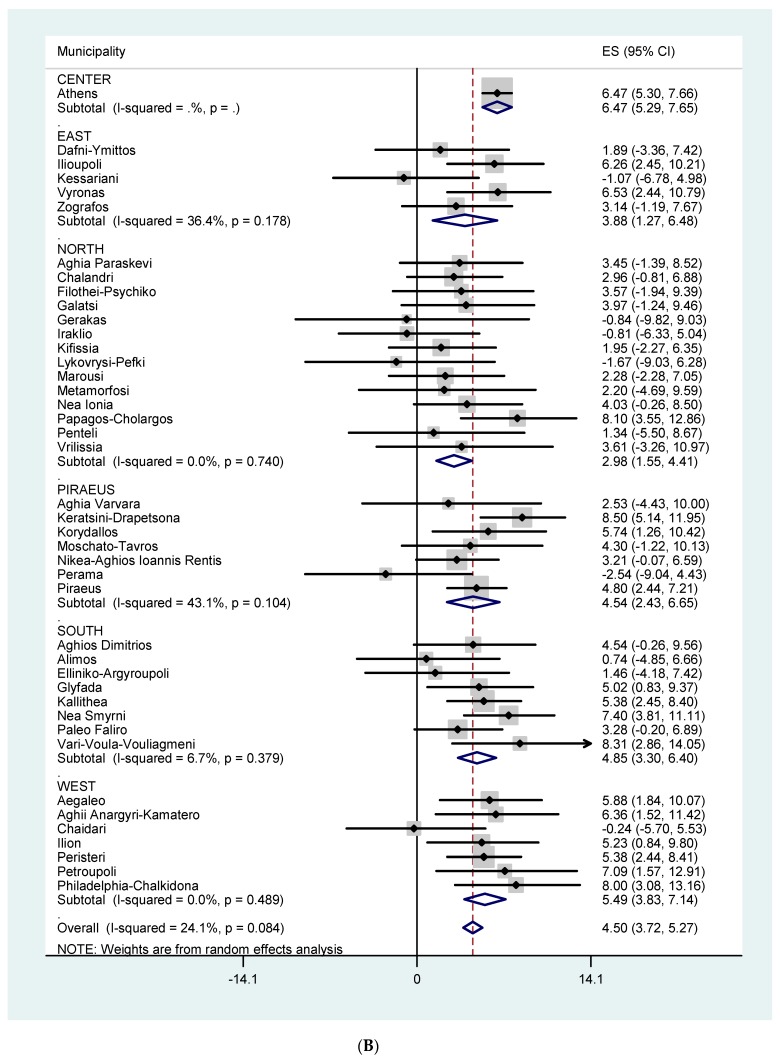
(**A**) % increase in cardiovascular mortality, all ages, per 1 °C increase in maximum temperature in each municipality, using Thissio measurements and threshold of 31.5 °C. (**B**) % increase in cardiovascular mortality among the elderly (75+) per 1°C increase in maximum temperature in each municipality, using Thissio measurements and threshold of 31.5 °C.

**Figure 5:**
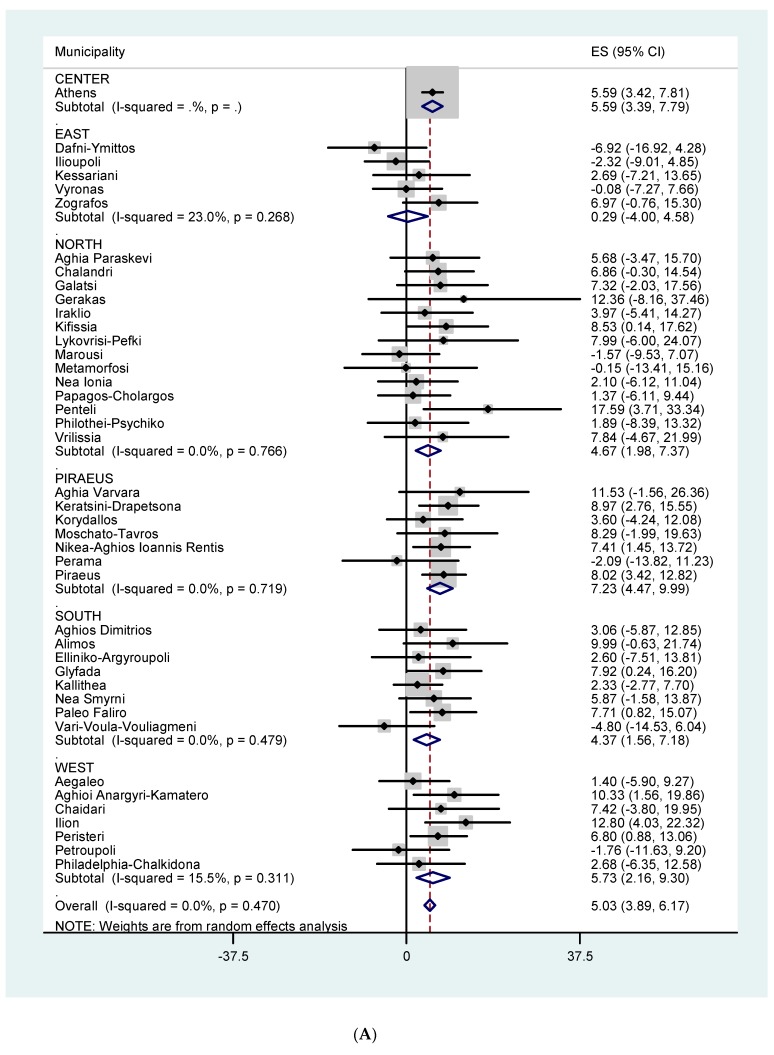

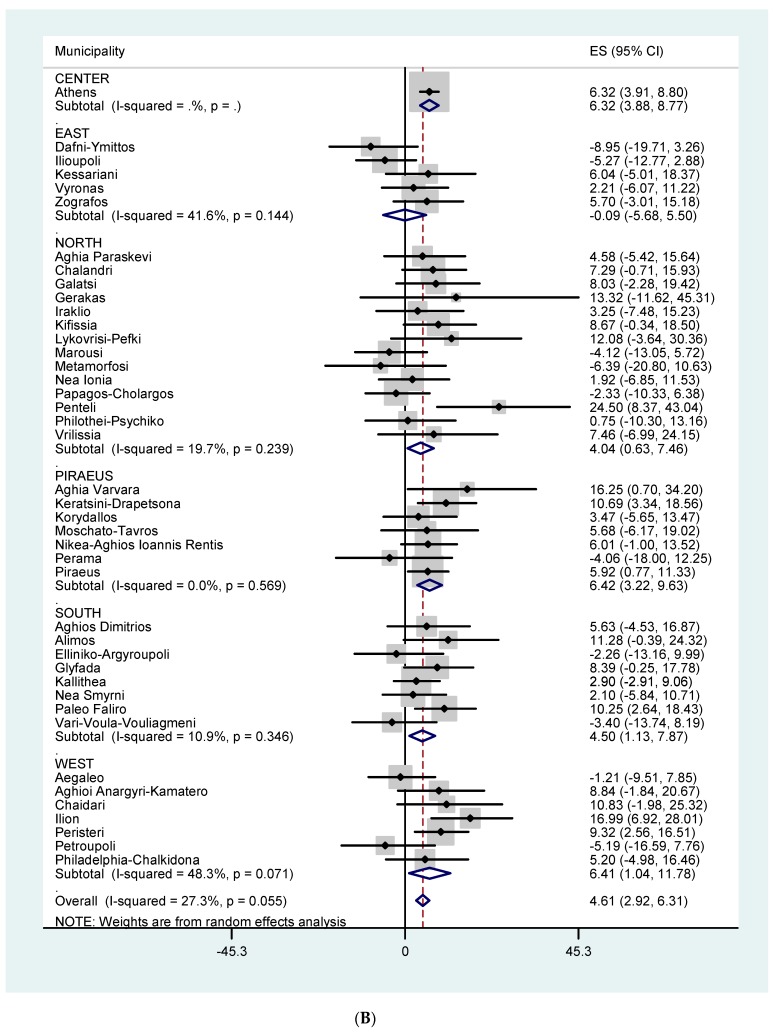
(**A**) % increase in respiratory mortality, all ages, per 1 °C increase in maximum temperature in each municipality, using Thissio measurements and threshold of 31.5 °C. (**B**) % increase in respiratory mortality among the elderly (75+) per 1 °C increase in maximum temperature in each municipality, using Thissio measurements and threshold of 31.5 °C.

Figure 3A,B show the effects of temperature on all-cause mortality for all ages and for those ≥ 75 years of age, respectively, by Municipality and by Sector. The effect estimates (for all ages) varied from −0.64 to 5.61. The overall heterogeneity in Municipality specific temperature effects was 39.8 % (*p* = 0.005) and 41.4 % (*p* = 0.003) for all ages and 75+, respectively. Figure 4A,B and Figure 5A,B show the effects of temperature on CVD mortality for all ages and for 75+ and respiratory mortality for all ages and for 75+, respectively, by Municipality and by Sector. The effect estimates varied from −2.74 to 6.86 for CVD mortality, all ages. The overall heterogeneity in Municipality specific temperature effects was 36.3 % (*p* = 0.011) and 24.1 % (*p* = 0.084) for all ages and 75+ respectively. The effect estimates varied from −6.91 to 17.60 for respiratory mortality, all ages. The overall heterogeneity in Municipality specific temperature effects was 0.0 % (*p* = 0.470) and 27.3 % (*p* = 0.055) for all ages and 75+ respectively.

Table 4 presents the percent increase in mortality per 1 °C increase in maximum daily temperature at the 25th and 75th percentile of the distribution of the identified (i.e., those with statistically significant meta-regression coefficient) effect modifiers. Percent area coverage by buildings, population density, total length of roads per km^2^ within Municipality, average of mean and maximum temperature, the percentage of population born outside the EU countries, unemployment and long-term unemployment rate and the percentage of early leavers from education significantly modified the effect. In contrast, area covered by green space (%), density of the road network within municipality (total road length in km per km^2^), total area (km^2^), youth unemployment rate (%), population aged 25–64 with lower secondary education attainment (%), population aged 25–64 with upper secondary or tertiary education attainment (%) did not significantly modify the effect of maximum temperature.

Supplementary Figures S2–S7 show the temperature effects on mortality by municipality, using the E-OBS dataset with sector-specific threshold and temperature data. As expected, there was generally less differentiation in temperature effects between municipalities using this approach, but the reduction was not consistently significant. In contrast to the reduction in the heterogeneity between effects on all and CVD mortality, there was a slight increase in the heterogeneity observed in respiratory mortality effects. The effect estimates of temperature increase per 1 °C above the threshold with this approach preserved the same pattern, i.e., they were larger for respiratory effects and larger for the elderly for CVD and all causes, but were generally smaller although still statistically significant. More specifically the effects on total natural mortality decreased by 34 % in the Athens center, Pireaus, the South and West sectors, and by 15 % and 21 % in the East and North sectors. CVD effect decreased by 34 to 45% in the Athens center, Pireaus, the South and West sectors and 27 % and 24 % in the other 2 sectors. For the effects on respiratory mortality, there was smaller decrease generally and even the effects on the East sector increased, probably due to the small counts of respiratory deaths leading to higher random variability. 

## 4. Discussion

We found significant effects of daily temperature increases on all-cause, cardiovascular, and respiratory mortality in the Greater Athens Area, based on estimated effects in each of the 42 Municipalities. However, heterogeneity in the effect estimates in the various Municipalities was observed in several instances and effect-modifying variables were identified. The effect was increased with increasing % area coverage of buildings, length of roads/km^2^, population density, average value of mean and maximum daily temperature and several SES variables. Due to the relatively small number of Municipalities, we only tested effect modification in models including the potential effect modifiers one by one. Therefore, it is possible that the effect modifiers are indicators of different SES associated with environmental and land use variables. In order to further examine the role of temperature, we alternatively used a modelled temperature estimate per Municipality as exposure and calculated the effects. We found that heterogeneity was somewhat reduced but not eliminated and the reduction was not entirely consistent among causes of death and age groups.

Few studies have investigated the within-city heterogeneity in the effects of temperature and its determinants in Europe, whilst there are more studies from North America and Asia. Xu et al. [35] applied an analysis by census tract in Barcelona contrasting “hot” and other days during the warm seasons of 1999–2006. They found an overall increase of 30 % on all-cause mortality on “hot” days and identified the percentage of old buildings, manual workers and residents perceiving little surrounding greenness as effect modifiers. In our analysis, we also identified effect modification by SES variables, and specifically, we found that larger temperature effects on mortality are associated with larger percentages of non-EU born individuals, higher unemployment rates and larger percentages of early leavers from education. These SES effect modifiers probably represent the general socioeconomic situation indicating that more deprived areas are associated with worst housing conditions and less opportunity to avoid the ambient heat. We did not find effect modification by the proportion of green space. Milojevic et al. [29] identified urban UHI hotspots in London and investigated whether heat-related mortality varied by UHI anomalies. They found little variation for heat-related mortality and concluded that there is relatively complete acclimatization of the population to the UHI effect. 

In studies in North America similar effect modifiers characterizing SES and urban planning have been identified [47,48]. In analyses of data in New York City Madrigano et al. [32] and Klein Rozenthal et al. [31] found that mortality during heat waves is larger in areas with lower SES and is also associated with other neighborhood characteristics. Also, they found that higher mortality was associated with areas characterized by higher surface temperature. Smargiassi et al. [27] also found that heat-related mortality is larger in areas with higher surface temperature. The latter finding is consistent with our finding of effect modification by the average of mean and maximum temperature per municipality. A similar finding is reported by Goggins et al. [26] in an analysis in three climatic zones within the city of Kaohsiung in Taiwan where heat-related mortality increase was identified in two out of three zones and Goggins et al. [25] in an analysis in Hong Kong where they report that UHI exacerbate the negative consequences of heat-related mortality. In Hong Kong data also indicate that heat-related effects are larger in vulnerable populations in terms of SES, age and other personal characteristics [24]. 

Our study has the advantage of being one of the very few studies in Europe to explore the intracity variations in heat-related deaths, in a large metropolitan area with Mediterranean climate and particularly vulnerable to future heat-related stress. However, the available climatic characterization at small spatial scale over the city area is based on a model for temperature, which probably over-smooths the temperature extremes and provides no information on other meteorological variables. Several studies have used land surface temperature from satellite data based on very few dates to characterize the spatial variability of temperature. This type of data incorporates as much measurement error or perhaps more compared to our model, because it also includes temporal error. Information on the mobility of the population would be very useful for our study. It would help to identify to what extent the inhabitants of an area are exposed to the temperature characterizing their area or are mobile in such a way that they are exposed to the conditions of various areas within a city. It may be inferred that at least the more elderly populations may not be so mobile. However, data on time-location-activity patterns are not available. 

## 5. Conclusions

In conclusion, the few studies on investigating the heterogeneity of within-city heat-related mortality follow various designs and are not easily comparable. Furthermore, various cities are characterized by different environmental conditions and climate, making local studies necessary. Despite the sparse results, it appears that there are SES, environmental, and personal determinants of the magnitude of heat-related effects on mortality, which are detected with some consistency and should be further investigated.

## Figures and Tables

**Table 1:** Temperature from the central fixed-site Thissio and from the E-OBS dataset, 1/04 to 30/09, 2000–2012.

	Greater Athens Area *	Sectors **					
		Athens Center	East	West	North	South	Piraeus
Maximum daily temperature (°C) Mean (SD)	29.1 (5.8)	27.7 (5.5)	27.2 (5.6)	26.2 (5.8)	26.1 (5.7)	27.4 (5.4)	26.8 (5.6)
Average daily temperature (°C) Mean (SD)	24.5 (5.3)	23.4 (5.1)	22.9 (5.2)	21.8 (5.3)	21.8 (5.3)	23.2 (5.1)	22.4 (5.2)

* based on measurements from the Thissio fixed-site. ** based on the E-OBS dataset.

**Table 2:** Population and daily number of deaths for the Greater Athens Area and by sector, 1/04 to 30/09, 2000–2012.

Area	Population	Daily number of deaths: mean (sd)								
		All ages			65–74 yrs			75+ yrs		
		All ^a^	CVD ^b^	Resp ^c^	All ^a^	CVD ^b^	Resp ^c^	All ^a^	CVD ^b^	Resp ^c^
**Greater Athens Area**	3193322	76.8 (12.4)	35.1 (7.4)	7.4 (3.2)	13.9 (4.2)	5.6 (2.6)	0.98 (1.02)	50.6 (10.3)	25.7 (6.2)	5.8 (2.8)
**Athens center**	664046	21 (5.1)	9.6 (3.5)	2.2 (1.5)	3.3 (1.9)	1.3 (1.2)	0.2 (0.5)	14.3 (4.3)	7.2 (2.9)	1.7 (1.4)
**East sector**	270573	6.4 (2.7)	2.8 (1.7)	0.6 (0.8)	1.1 (1.1)	0.4 (0.7)	0.1 (0.3)	4.3 (2.2)	2.1 (1.5)	0.5 (0.8)
**West sector**	498681	10.3 (3.6)	4.7 (2.3)	0.9 (1.0)	2.1 (1.5)	0.8 (0.9)	0.1 (0.4)	6.4 (2.9)	3.3 (1.9)	0.7 (0.9)
**North sector**	706250	13.3 (4.0)	6.0 (2.5)	1.3 (1.2)	2.3 (1.5)	0.8 (0.9)	0.1 (0.4)	9.0 (3.2)	4.5 (2.1)	1.0 (1.1)
**South sector**	537812	12.2 (3.9)	5.6 (2.5)	1.1 (1.2)	2.2 (1.5)	0.9 (1.0)	0.1 (0.4)	8.1 (3.2)	4.1 (2.1)	0.9 (1.0)
**Piraeus sector**	515960	13.6 (4.1)	6.4 (2.7)	1.3 (1.2)	2.7 (1.7)	1.1 (1.1)	0.2 (0.5)	8.5 (3.3)	4.5 (2.3)	1.0 (1.0)

^a^: all natural causes, ICD10:A00-R99. ^b^: CVD cardiovascular causes; ICD10: I00-I99. ^c^: Respiratory causes; ICD10: J00-J99.

**Table 3:** Percent increase in mortality and associated 95 % confidence interval per 1 °C increase in maximum temperature (average of lags 0–3) above the turning point (31.5 °C), by cause of death and age group in the Greater Athens Area.

Cause of Death	Age Group		
	All Ages	65–74 Years	75+ Years
**All causes ^a^**	4.16 (3.73, 4.60)	2.87 (1.95, 3.80)	5.17 (4.65, 5.69)
**CVD ^b^**	5.34 (4.74, 5.93)	2.76 (1.32, 4.22)	6.17 (5.49, 6.86)
**Respiratory ^c^**	5.90 (4.57, 7.24)	7.68 (4.39, 11.08)	5.89 (4.42, 7.38)

^a^: all natural causes, ICD10:A00-R99. ^b^: CVD cardiovascular causes; ICD10: I00-I99. ^c^: Respiratory causes; ICD10: J00-J99.

**Table 4:** % increase in daily number of deaths per 1 °C increase in maximum daily temperature, at the 25th and 75th percentile of the distribution of identified effect modifiers, by cause of death.

Effect Modifier	Value of 25th–75th Percentile	All Causes-All Ages		All Causes-75+ Yrs		CVD Causes-All Ages	
		% increase at the 25th perc.	% increase at the 75th perc.	% increase at the 25th perc.	% increase at the 75th perc.	% increase at the 25th perc	% increase at the 75th perc
**% area coverage by buildings**	11.4–30.5	1.9 (1.3–2.6)	3.2 (2.7–3.7)	-	-	2.6 (1.7–3.6)	4.5 (3.8–5.2)
**Length of roads/km^2^**	10.3–23.6	2.1 (1.4–2.7)	3.0 (2.5–3.6)	-	-	2.7 (1.7–3.7)	4.3 (3.6–5.1)
**Population density, inhabitants/km^2^**	5244–12953	1.9 (1.4–2.5)	3.2 (2.9–3.6)	2.9 (2.1–3.7)	3.9 (3.3–4.5)	2.7 (1.8–3.5)	4.4 (3.8–4.9)
**Mean value of max temperature, °C**	26.1–27.2	2.1 (1.4–2.8)	3.0 (2.5–3.6)	-	-	3.0 (2–3.9)	4.3 (3.5–5)
**Mean value of mean temperature, °C**	21.8–22.9	-	-	-	-	3.0 (2.0–3.9)	4.3 (3.5–5.0)
**Population born in non EU28 countries (%)**	6.5–10.5	2.1 (1.5–2.7)	2.9(2.5–3.3)	2.9 (2.1–3.7)	3.7 (3.1–4.3)	3.0 (2.1–3.9)	3.8 (3.2–4.5)
**Unemployment rate (%)**	13.9–19.7	2.1 (1.5–2.7)	3.1(2.6–3.6)	2.9 (2.1–3.7)	4.0 (3.3–4.7)	-	-
**Long-term unemployment rate (12 months and more, %)**	10.4–14.6	2.2 (1.6–2.8)	3.0(2.5–3.6)	3.0 (2.2–3.8)	3.9 (3.2–4.6)	-	-
**Early leavers from education and training (%)**	2.8–6.5	-	-	3.0 (2.2–3.7)	3.9 (3.2–4.6)	-	-

## Supplementary Materials

The following are available online at https://www.mdpi.com/1660-4601/16/19/3689/s1. Figure S1: Temperature-mortality association by sector, using the E-OBS data, Figure S2: % increase in total mortality, all ages, per 1 °C increase in maximum temperature in each municipality, using EOBS data and threshold of each sector, Figure S3: % increase in total mortality among elderly (75+) per 1 °C increase in maximum temperature in each municipality, using EOBS data and threshold of each sector, Figure S4: % increase in cardiovascular mortality, all ages, per 1 °C increase in maximum temperature in each municipality, using EOBS data and threshold of each sector, Figure S5: % increase in cardiovascular mortality among elderly (75+) per 1 °C increase in maximum temperature in each municipality, using EOBS data and threshold of each sector, Figure S6: % increase in respiratory mortality, all ages, per 1 °C increase in maximum temperature in each municipality, using EOBS data and threshold of each sector, Figure S7: % increase in respiratory mortality among elderly (75+) per 1 °C increase in maximum temperature in each municipality, using EOBS data and threshold of each sector.
