# Supplementary material for: Spatial Variability in the Effect of High Ambient Temperature on Mortality: An Analysis at Municipality Level within the Greater Athens Area
Source: Int J Environ Res Public Health. 2019 Sep 30;16(19):3689. doi: 10.3390/ijerph16193689 (PMC6801795; doi:10.3390/ijerph16193689)
Supplement: Supplementary file 1 [file ijerph-16-03689-s001.pdf]

Supplementary Materials

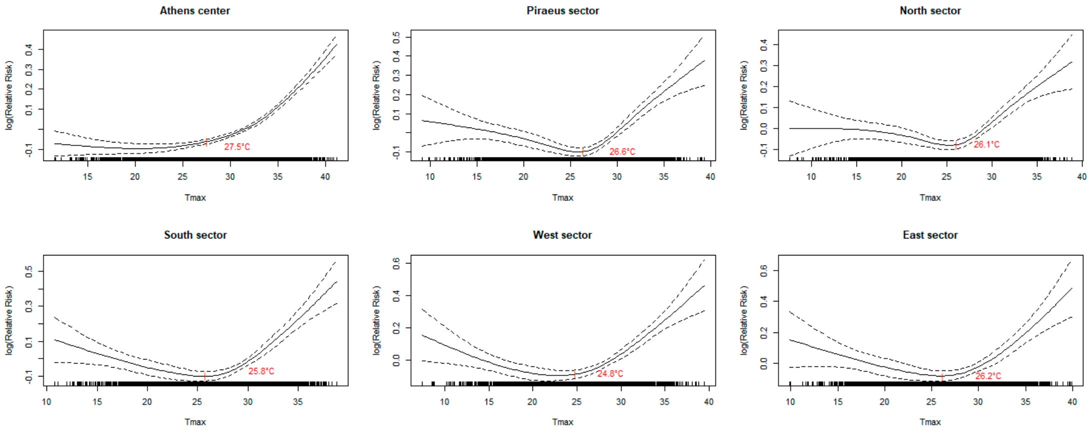

Figure S1. Temperature-mortality association by sector, using the E-OBS data.

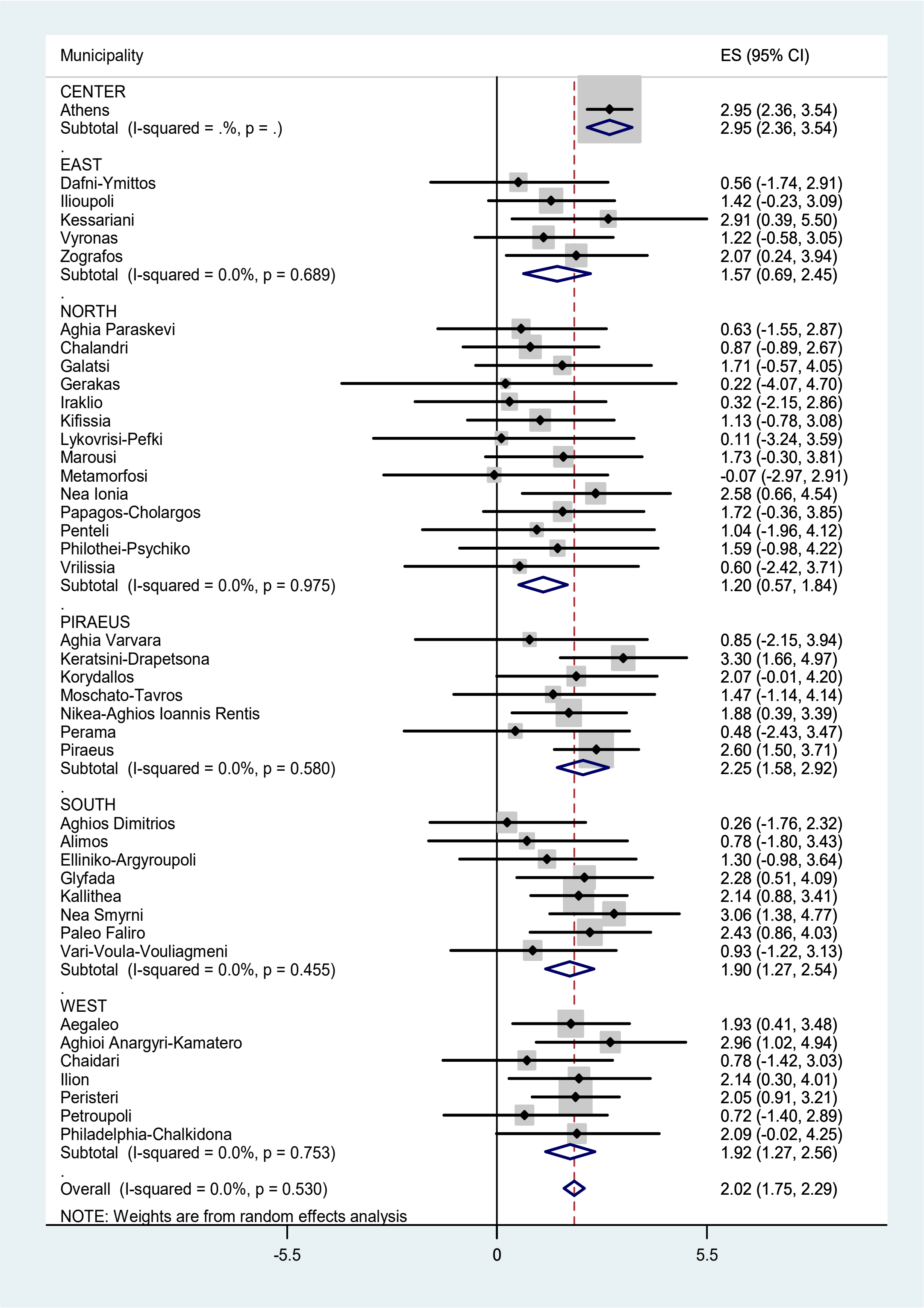

Figure S2. % increase in total mortality, all ages, per 1 °C increase in maximum temperature in each municipality, using EOBS data and threshold of each sector.

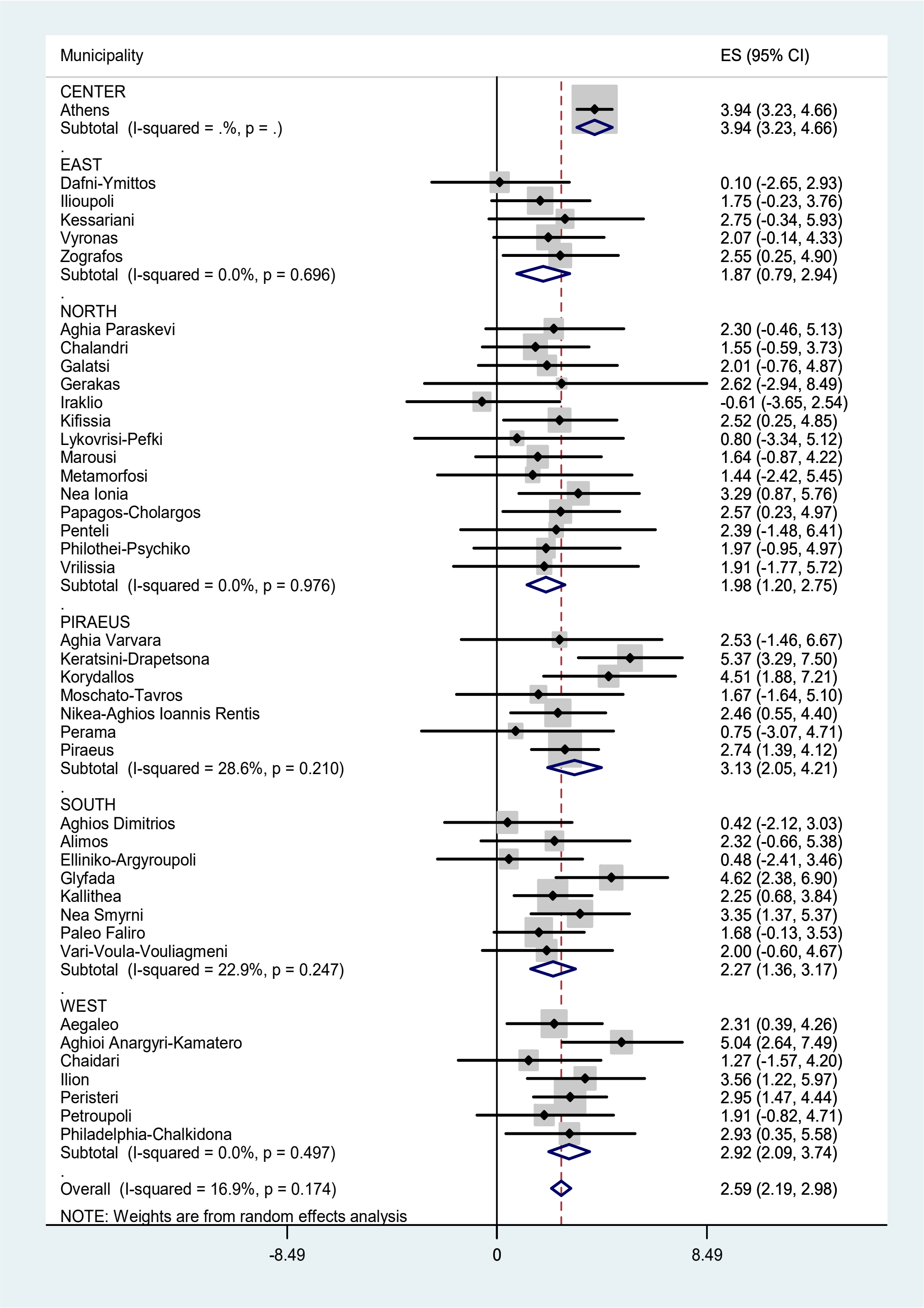

Figure S3. % increase in total mortality among elderly (75+) per 1 °C increase in maximum temperature in each municipality, using EOBS data and threshold of each sector.

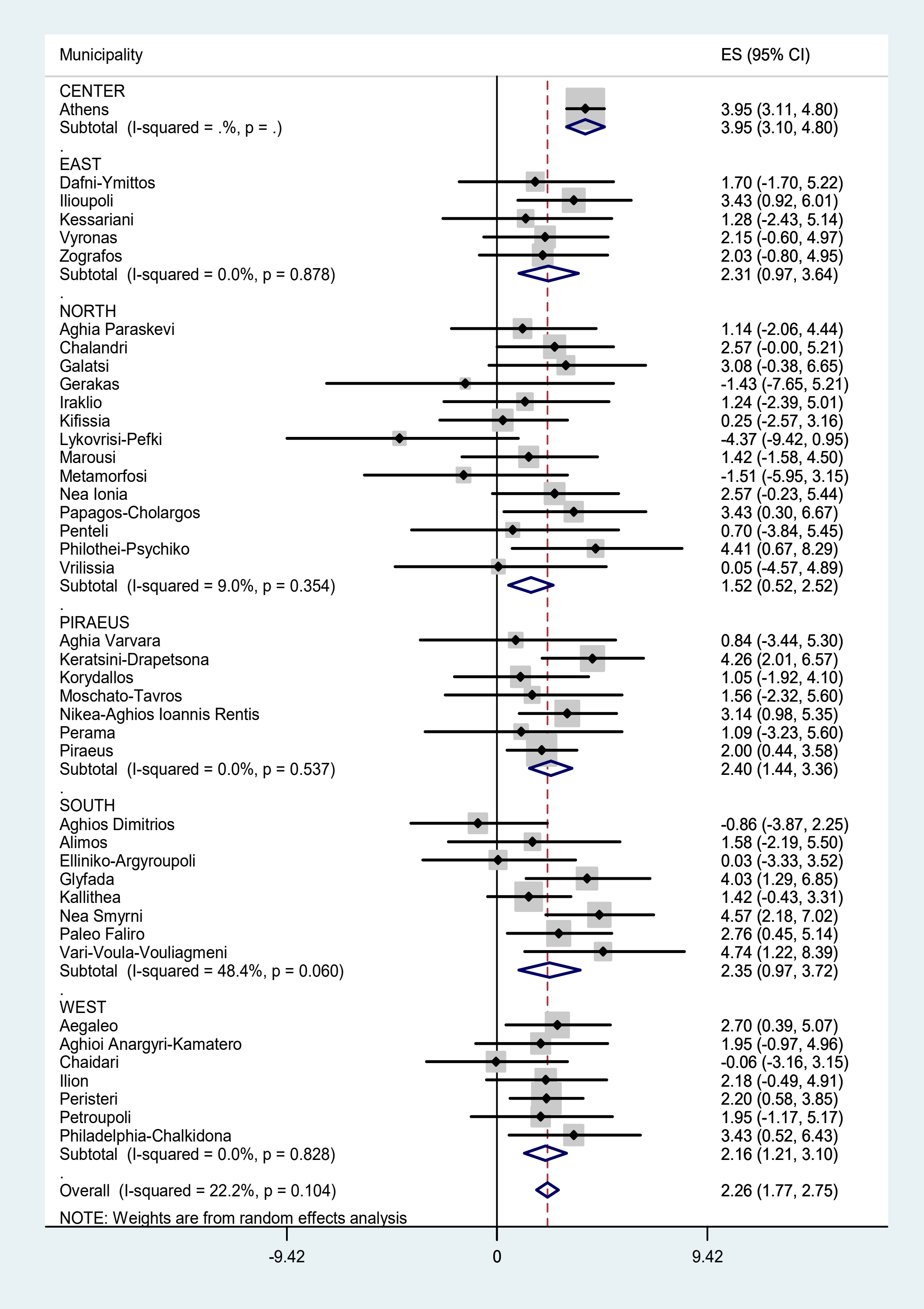

Figure S4. % increase in cardiovascular mortality, all ages, per 1 °C increase in maximum temperature in each municipality, using EOBS data and threshold of each sector.

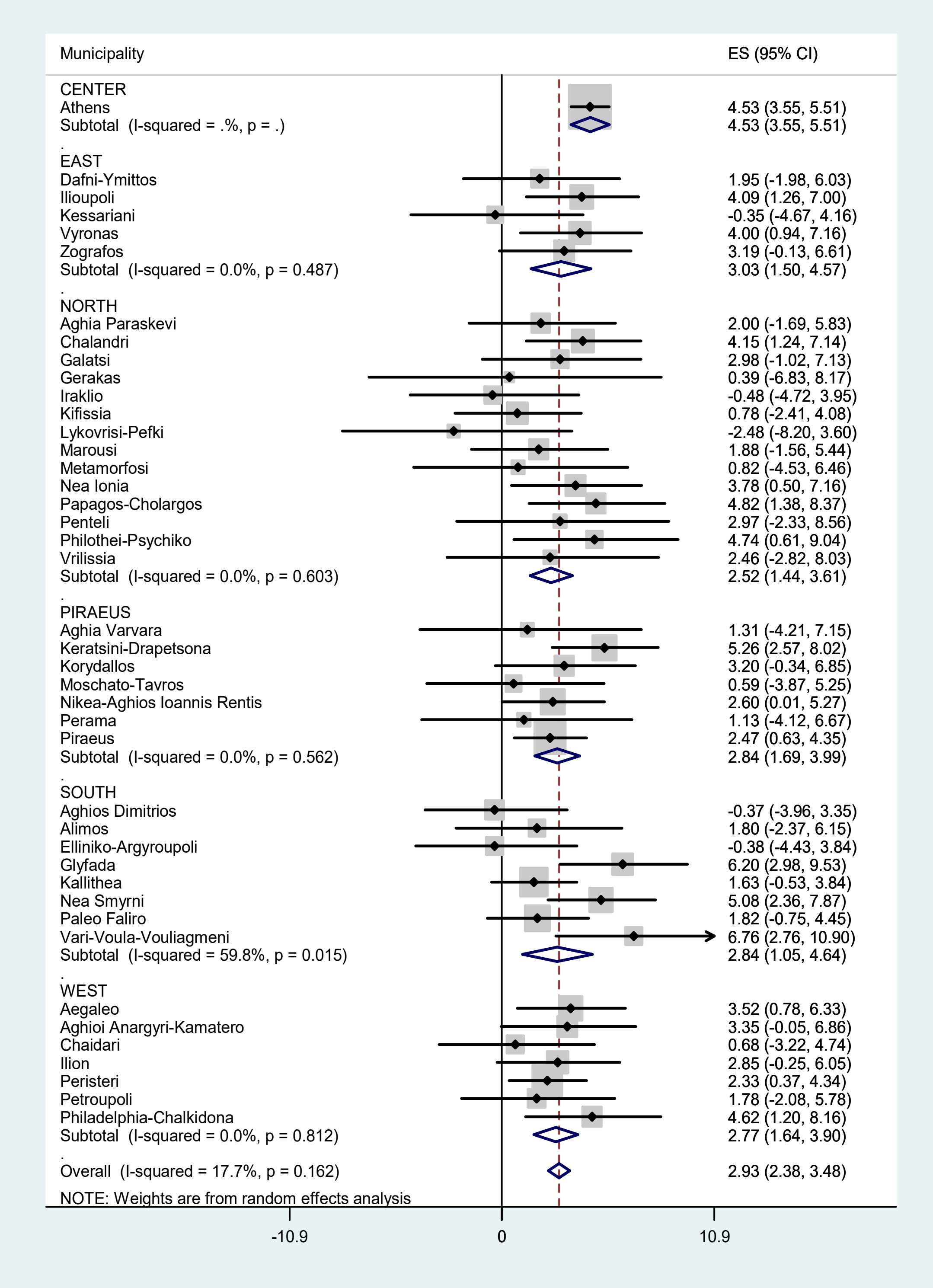

Figure S5. % increase in cardiovascular mortality among elderly (75+) per 1 °C increase in maximum temperature in each municipality, using EOBS data and threshold of each sector.

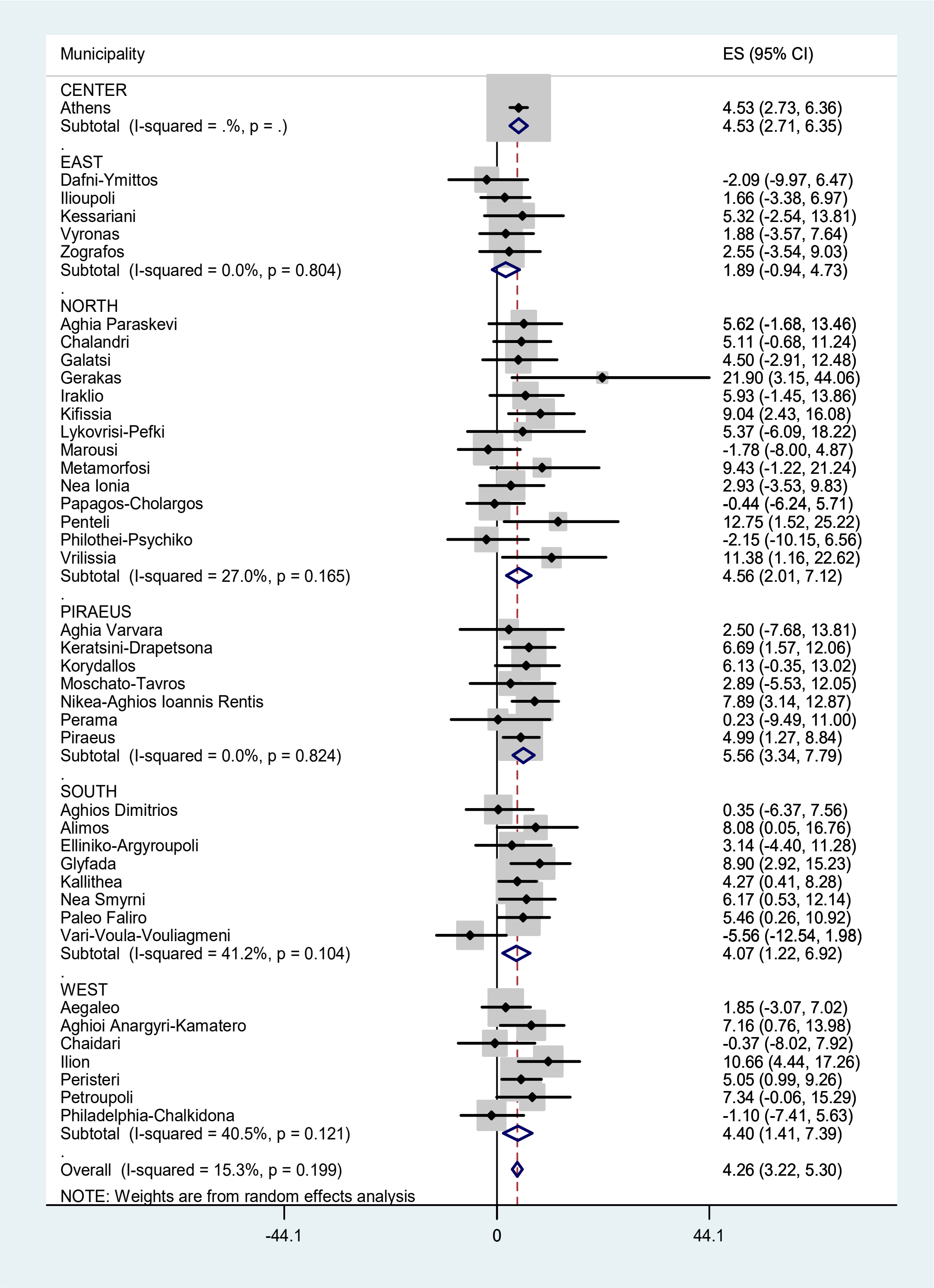

Figure S6. % increase in respiratory mortality, all ages, per 1 °C increase in maximum temperature in each municipality, using EOBS data and threshold of each sector.

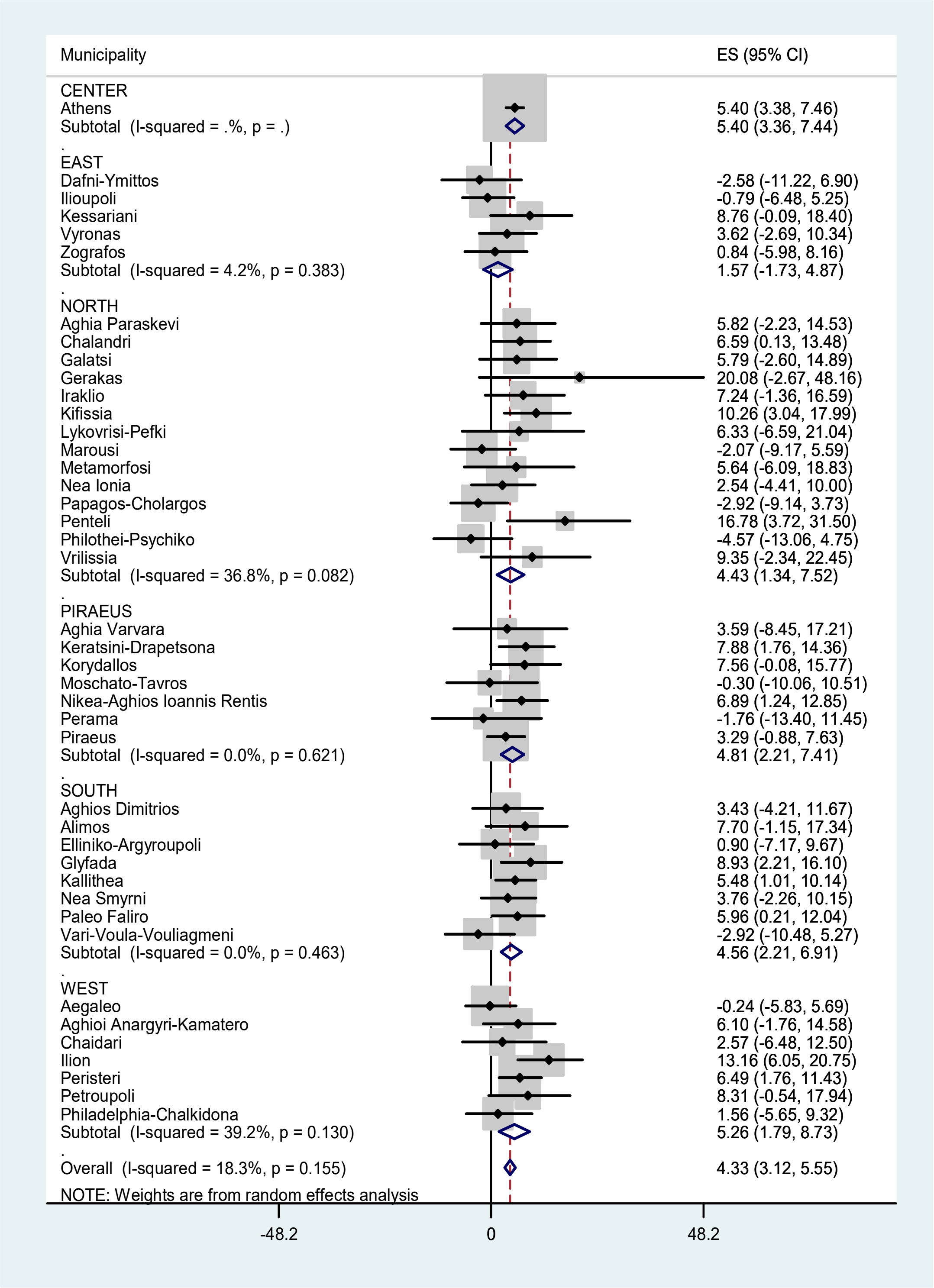

Figure S7. % increase in respiratory mortality among elderly (75+) per 1 °C increase in maximum temperature in each municipality, using EOBS data and threshold of each sector.
